# Semaglutide and human reproduction: caution at the intersection of energy balance, ovarian function, and follicular development

**DOI:** 10.1186/s12958-025-01435-7

**Published:** 2025-08-08

**Authors:** E. Scott Sills, Conor Harrity, Howard I. Chu, Jing-Wen Wang, Fan Yang, Samuel H. Wood

**Affiliations:** 1 Pelican Bay Biology Group, Center for Advanced Genetics, Crescent City, CA USA; 2 Experimental Science Systems, Harriman, TN USA; 3 Department of Obstetrics & Gynaecology, Royal College of Surgeons in Ireland, Dublin, Ireland; 4 Department of Psychobiology, University of California-Los Angeles, Los Angeles, CA USA; 5 Gen 5 Fertility Center, San Diego, CA USA; 6 Fertility Center, 3420 Carmel Mountain Road, Suite 200, San Diego, Calif 92121 USA

**Keywords:** Semaglutide, GLP-1, Insulin, Ovary, Reproduction

## Abstract

Obese or overweight patients considering IVF are generally counselled to reduce weight closer to target BMI (i.e., < 30 kg/m^2^) by interventions entailing dietary change with a structured exercise program. There is little disagreement that supervised weight loss can improve reproductive outcome when successful, although there are refractory cases where weight goals are unmet. Because low-grade chronic inflammation and altered immune function are characteristic of obesity and antagonize implantation, any pre-IVF weight loss facilitated by semaglutide (SG) would be helpful. However, no preclinical data have considered the ovarian implications of SG. Several formulations of SG are now available to assist in chronic weight management, treatment of type-2 diabetes, or both. SG is 31-amino acid lipopeptide with action at the glucagon-like peptide-1 (GLP-1) receptor, which augments insulin secretion while lowering hepatic glucagon output. SG thus enters a multiorgan network where insulin, AMP-activated protein kinase (AMPK), insulin-like growth factor-1 (IGF-1), mammalian target of rapamycin (mTOR), and sirtuin pathways manage ambient nutritional conditions. As GLP-1 directly influences insulin release and curtails satiety, SG adjusts many biochemical cascades where potential interference with oocyte development or embryo/endometrial crosstalk require clarification. Particularly if used outside manufacturer’s guidance (i.e., for aesthetic or personal reasons), SG could bring unwelcome challenges to fertility clinics where obesity and dyslipidemia are merely exchanged for the new problems of starvation and sarcopenia. Here we examine known GLP-1 actions where energy balance, ovarian aging, and oocyte competence converge; off label SG use should be avoided until its signaling effects throughout the reproductive axis are more carefully studied.

## Introduction

Current national survey data show age-adjusted prevalence of severe (Class III) obesity among U.S. adults is just under 10%. Because overweight and obesity affect women more than men [1], the problem—and its treatments—cannot be ignored in clinical reproductive practice. While the overweight challenge has yet to be systematically investigated specifically in an assisted reproductive setting, single-center data are concerning. One study of in vitro fertilization (IVF) patients in Ecuador (*n* = 191) reported frequency of overweight and obese females at 45% and 19.9%, respectively [2]. Another sample from USA (*n* = 2069) found 49% of IVF patients had body mass index (BMI) 30-34.9, 26% had BMI 35-39.9, while 7% had a BMI > 50 [3]. It is possible that milder obesity cases may be beneficially managed with dietary counseling alone. For example, when nutritional features were tracked in infertile women (*n* = 300) and healthy fertile controls (*n* = 300), diets with high glycemic index, glycemic load, and dietary inflammatory index were more often associated with infertility [4]. Unsurprisingly, such patients typically have a more guarded prognosis than age-matched, normal weight peers, since obesity is accompanied by an increased pregnancy loss rate. This means that while obese patients may initially conceive by IVF, many such pregnancies will miscarry and not continue to term [5]. The higher observed miscarriage rate in obesity could be due to decreased oocyte quality, altered endometrial receptivity, or both [6]. The arrival of glucagon-like peptide-1 (GLP-1) modulators thus has met both caution and welcome in reproductive endocrinology, where effective adjuncts to weight control are urgently needed.

### GLP-1, energy status, and reproduction

Weight loss occurs only when energy expenditure surpasses caloric input [7], and GLP-1 agents can approximate, if not surpass, results once possible only with invasive bariatric procedures [8, 9]. Nevertheless, using anti-obesity medications by overweight or obese patients requires awareness of sweeping endocrine effects [10] including how changes in cellular energy may cascade into reproductive biology. Ovarian capacity often declines earlier than other organs in normal aging [11]. Extremes in energy balance are considered harmful; either very low or very high adiposity impairs fertility. For obesity, its default state of low-grade, chronic inflammation and disordered immune function [12] severely lowers nicotinamide adenine dinucleotide (NAD+) at cellular, tissue, and systemic levels, leading to restricted cellular energy production [13].

Sustained energy shortage cues AMP-activated protein kinase (AMPK) to drive follicular atresia [14]. AMPK, a master regulator of energy homeostasis, induces autophagy in a dose-dependent manner. It also acutely inhibits the stimulatory effects of luteinizing hormone (LH)/protein kinase A (cAMP-dependent protein kinase) on progesterone production in luteal cells, an effect reversed by exogenous cholesterol [14]. AMPK is directly involved in human granulosa cell steroid production and other processes often disrupted in cell adhesion, lipid metabolism, and inflammation. Silencing AMPK promotes follicle growth accompanied by increased serum anti-Müllerian hormone (AMH) [15]. Importantly, the follicle-stimulating hormone receptor (FSHR)-mammalian target of rapamycin (mTOR)- hypoxia-inducible factor 1 (HIF1) pathway helps follicles avoid atresia for the duration of any local environmental stress [16].

The local nutritional climate is mediated by mTOR to regulate cell growth, differentiation, metabolism, and autophagy [17]. mTOR contributes to more than 700 different phosphorylation reactions including the tuberous sclerosis/TSC1 (hamartin)-TSC2 (tuberin) complex, which itself dampens the mTOR/S6K/4E-BP pathway (i.e., ribosomal protein S6 kinase 1/S6K and eukaryotic translation initiation factor 4E-binding protein/4E-BP) [18]. These imbricated systems remain incompletely mapped [19]. In mammalian reproduction, mTOR guides follicular recruitment, influences oocyte maturation in meiosis, coordinates signaling to granulosa/theca units, orchestrates puberty onset and influences ovarian aging [17, 20]. Reduced ‘silent mating type information regulation 2 homologue 3’ (SIRT3) in experimental oocytes increases mitochondrial reactive oxygen species (ROS) production, leading to impaired embryo competence and development [21].

NAD + and its reduced form (NADH) are coupled metabolites facilitating key redox reactions. They enable energy transfer through glycolysis and mitochondrial respiration to support cell growth and survival. Numerous regulatory enzymes broadly involved in cell functions require NAD + as a co-substrate for catalytic activity [22]. Improving intraovarian NAD + levels in aging mice can amplify the follicle pool, ovulation potential, and livebirth rate. Replenishing NAD + with nicotinamide riboside reduces ROS levels and decreases spindle anomalies in older oocytes, while raising mitochondrial transmembrane potential with decreased mitochondrial clustering [23]. Similar results were obtained in a murine model by nicotinamide mononucleotide (NMN), which enhanced oocyte meiotic competency by suppressing apoptosis, maintaining normal spindle/chromosome structure, and conservation of ovastacin—a cortical granule component [24].

### Evidence and rationale for weight optimization

Clinical research has shown overweight and obese women experience high intracellular lipid toxicity, more inflammation, and greater oxidative stress which negatively impacts health generally and reproductive function specifically [2]. Adipose tissue serves an endocrine role by contributing to steroid output, carbohydrate control, and inflammatory signaling [25]. Its pleiotropic role is typified by synthesis and release of numerous adipokines with diverse and extensive signaling effects [26]. Although fat deposits can develop directly in mammalian ovarian tissue from pharmacologic blocking of steroidogenic synthesis and stromal lipid accumulation [27], this is neither common nor necessary to compromise ovarian function.

Health-promoting lifestyle programs are essential in the assisted reproductive technologies to optimize pregnancy and livebirth rates [28]. One recent study of infertile overweight or obese women scheduled for IVF (*n* = 2381) reported that a 60-day weight reduction intervention before treatment can increase neonatal birth weight, reduce maternal blood glucose concentration, and improve maternal insulin resistance [29]—all desirable goals prior to ovulation induction via gonadotropins.

A ‘first meal’ dose of β-glucan or arabinoxylan extract followed 6 h later by a 20% glucose solution (second meal) suppressed blood glucose elevation. Both arabinoxylan and β-glucan increased the levels of short-chain fatty acids in ileum and cecum, respectively. Of note, GLP-1 secretion in the blood increased with β-glucan and showed an increasing trend with arabinoxylan, suggesting barley β-glucan and arabinoxylan are fermented in the intestinal tract to favor endogenous GLP-1 release [30], thus mimicking SG effects. This was consistent with murine research which confirmed improved glucose tolerance after high β-glucan barley intake associated with a subsequent rise in GLP-1 secretion [31].

Several investigators have established a connection between dietary restriction and mitochondrial activity [32, 33]. The sirtuin-1 (SIRT1)-AMPK system processes metabolic information to increase number and function of mitochondria [34, 35] similar to resveratrol [36, 37] and metformin [38, 39]. A reaction to calorie restriction has been experimentally shown to affect lifespan in animals [40, 41] and humans [42, 43]. For example, rodents on severely restricted diets can suffer fertility impairment or total loss of reproductive capacity [44, 45]. In clinical practice, females underweight from profound dietary restriction likewise show lower fertility with endocrine features congruent with ovarian insufficiency [46]. However, in certain contexts limiting caloric intake is accompanied by desirable effects including an extension of ovarian operational capacity beyond expected limits. In nematodes, transient diet scarcity blunts reproduction by inducing latency in germline stem cells, a reversible process which is undone once ambient nutritional factors improve [47, 48]. Experimental studies have described how adversity can sharply attenuate reproductive success while defending germline reserves, with recovery of fertility when environmental conditions are more favorable [49, 50]. In many avian species, periodic molting to increase egg lay is attained commercially by programmed limitation of nutrients. Although induced molting is sometimes viewed as harmful or inhumane, it is known that wild birds do not eat despite plentiful food in nature. This biological process is especially evident in geese which may be anorexic for many weeks, and in king penguins where fasting can extend up to six months [51].

Intermittent fasting can likewise improve health and extend lifespan, but any superiority over caloric restriction has yet to be proven. In a murine model, improved oocyte quality was achieved with every-other-day fasting after one month [52]. Not only was antral follicle count and ovulation improved, both nuclear and cytoplasmic maturation were enhanced after this intervention. Of note, single-cell transcriptome analysis found the beneficial impact of intermittent fasting was mediated by restored NAD+/SIRT1-mediated antioxidant defenses [52]. These results were consistent with earlier murine data [53] where calorie restriction was noted to extend lifespan by increasing cellular NAD+/NADH ratio, promoting expression of peroxisome proliferator-activated receptors, and activating SIRT1 [53]. In human fibroblast Hs68 cells, calorie restriction has extended lifespan via boosted expression of nicotinamide phosphoribosyltransferase (NAMPT), intracellular NAD(+) levels, and SIRT1 action [54].

While some have noted the lack of evidence to support long-term calorie restriction [55], there is disagreement here. For example, when the growth hormone (GH)/Insulin like growth factor (IGF)-1 axis and insulin resistance were measured after intermittent fasting and caloric restriction over two years in non-diabetic obese subjects, metabolic parameters were positively impacted by caloric restriction, while intermittent fasting (with no caloric restriction) enhanced cellular resistance to disease but without weight loss [56].

It appears that moderate dietary restriction, can enhance ovarian function in mammals [57], although exactly how this works is not known. One hypothesis suggests metabolic resources are shunted to somatic maintenance which is prioritized over reproduction, where dietary scarcity temporarily favors pro-longevity processes [49]. The assessment may be incomplete since trade-offs between reproduction and lifespan during caloric restriction are not always easy to define [49].

### GLP-1 as a metabolic “conductor”

Underscoring the broad dispersal of energy management tasks throughout cell populations, GLP-1 can contribute to any of the above biosystems where nutritional status directs a physiological process. Nutritional stress likewise influences longevity [58, 59] and recent work has given a sharper picture of these interconnected networks [57, 60]. GLP-1 is sufficiently critical to overall life function that no true GLP-1 deficiency state has ever been documented. And because AMPK, IGF-1, mTOR, and the sirtuin pathways all interlock with GLP-1, the relevance of SG to reproduction rightly draws notice.

GLP-1 is a highly conserved regulator of energy homeostasis with almost complete sequence homology across many mammalian species [61]. Classified first as an incretin, GLP-1 protects against carbohydrate overload by enhancing insulin secretion and inhibiting glucagon secretion [62]. Since GLP-1 actions are intimately connected to insulin balance, SG cuts across innumerable dynamic biochemical systems with relevance to overall ovarian function and follicle development [63, 64]. While insulin is the chief regulator of lipid and glucose metabolism, IGF-1 is an organizer of growth and development. They can co-bind to highly homologous receptors which share close structural similarity and have common distal signaling pathways [65], exemplified by their joint initiation of network signaling to promote activation of maturation-promoting factor (MPF), catalyzing oocyte entry into M-phase of meiosis I and II [66].

Processes known to be mediated by GLP-1 have enlarged and diversified over time; its receptor in endometrium may fluctuate during the menstrual cycle [67, 68]. GLP-1 appears to activate calmodulin-dependent protein kinase II via calcium influx at L-type voltage-gated calcium channels [69]. Additionally, GLP-1 alters interleukin (IL)-6 to modulate hyperglycemia-induced apoptosis and endoplasmic reticulum degradation from homocysteine excess [70], an effect apparently independent of insulin dynamics [71].

Transactivation has been suggested for other receptors, including GLP-1 dampening of K^+^ channels and binding to epidermal growth factor receptors, leading to activation of phosphatidylinositol 3-kinase (PI3K) [72] followed by inhibition of apoptosis-related transcription factors [62, 73]. Interestingly, although not yet observed in humans, the active transcriptional response to GLP-1/Notch signaling is evident in the germline stem cell pool in *C. elegans* [74].

### Semaglutide: considerations before IVF

Semaglutide (SG) is a glucagon-like peptide-1 (GLP-1) receptor agonist which received U.S. FDA approval in 2021 (Wegovy^®^, Novo Nordisk; Plainsboro, New Jersey) for chronic weight management in adults. It is administered as a weekly 2.4 mg subcutaneous injection [75]. Ozempic^®^ (Novo Nordisk) and Rybelsus^®^ (Novo Nordisk) are other SG formulations approved for type 2 diabetes, but not for weight loss. They improve glucose handling by boosting pancreatic insulin secretion while dampening hepatic glucagon release [76]. SG also modulates neuronal pathways to stimulate satiety and energy expenditure while reducing energy intake [77].

When combined with lifestyle changes of dietary modification and a structured exercise program, SG can provide beneficial results for adults with BMI ≥ 30, or BMI of at least 27 plus diabetes, hypertension, dyslipidemia, obstructive sleep apnea, vascular disease, or history of myocardial infarction [78]. Off-label use of SG for aesthetic or personal reasons among non-obese patients is growing [79, 80] and can be expected to elicit similar—but not yet adequately studied— responses as produced in overweight or obesity.

Specifically, SG is thought to facilitate autophagy via enhanced AMPK [81]. It also boosts IGF-1-mediated muscle protein synthesis, inhibits NF-κB-mediated ubiquitin-proteosome degradation, and directly augments heat-shock factor-1-mediated myogenesis in myocytes [82]. Insulin signaling and its associated AMPK pathway are enhanced by SG [83]. NK cell impairment is common in obesity, a disorder which is substantially rectified with SG by its apparent enhancement of the CD98-mTOR-glycolysis axis [84]. While SG may decrease expression of NF-κB, TNF-α and IL-1β in some cell populations [85], this has not been observed specifically in ovarian tissue.

**Fig. 1:**
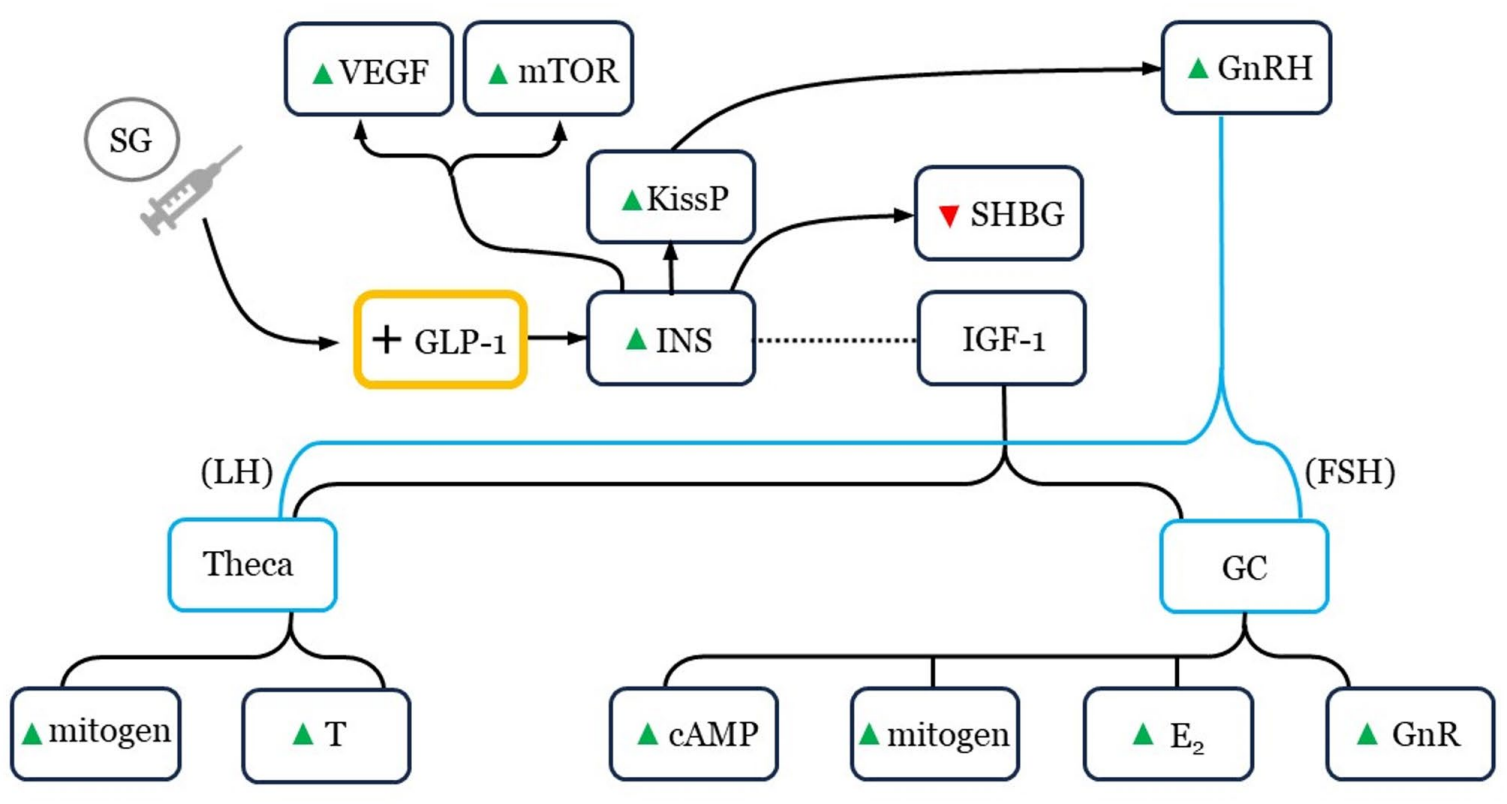
Schematic of selected direct and secondary semaglutide (SG) responses related to insulin (INS) and insulin-like growth factor 1 (IGF-1), with emphasis on central and peripheral reproductive and proliferative effects (blue). IGF-1 can potentiate oocyte maturation via phosphoinositide-3-kinase/v-akt murine thymoma viral oncogene homolog (PI3K/AKT) signaling. VEGF = vascular endothelial growth factor; mTOR = mammalian target of rapamycin; GnRH = gonadotropin hormone-releasing hormone; KissP = kisspeptin; SHBG = sex hormone binding globulin; GLP-1 = glucagon-like peptide 1; LH = luteinizing hormone; FSH = follicle stimulating hormone; GC = granulosa cell; T = testosterone; cAMP = cyclic adenosine monophosphate; E_2_ = estradiol; GnR = gonadotropin receptors

Signal inputs necessary for ovarian folliculogenesis are numerous, and how SG interfaces with transforming growth factor-beta 1, growth differentiation factor-9 (GDF-9), vascular endothelial growth factor (VEGF), and insulin-like growth factor-1 (IGF-1) awaits full characterization (see Fig. 1).

Platelet-derived growth factor and bone morphogenic proteins also appear central to cell migration, vascular support, and general ovarian function [86]. When cell exomes releasing these substances are inserted near undifferentiated oocyte stem precursors in an experimental ovarian insufficiency model, follicle development and restored reproductive capacity are observed [87]. How SG influences the function of these mediators remains to be studied in an ovarian setting.

Adverse but rare events associated with SG may include hypoglycemia, gastrointestinal upset, pancreatitis and pancreatic cancer, thyroid cancer, gallbladder events, or acute kidney injury [88]. Its packaging features a ‘black box’ warning for possible development of thyroid C-cell tumors, and the medication is contraindicated for those at risk for medullary thyroid cancer or type 2 multiple endocrine neoplasia. In addition, a history of acute pancreatitis is a relative contraindication [89].

More recently, an agent which targets both GLP-1 and GIP receptors (tirzepatide) received regulatory clearance for treatment of type 2 diabetes. It shares a similar mechanism of action with SG, and this dual receptor co-agonist affinity of tirzepatide may limit gastrointestinal symptoms [90].

SG is relatively long-acting with a half-life of about seven days when injected subcutaneously [91]. No formal guidance has been issued for a dosing schedule before or during fertility treatment. Because some data suggest possible harm to offspring, SG should be avoided in pregnancy or breastfeeding [10]. Of note, accidental SG use in pregnancy was reported in a diabetic patient (age 43yrs) where no adverse events were documented in mother or baby following intrapartum exposure of six months [92]. When used in advance of a programmed IVF cycle, discontinuation of SG for least 8 weeks before planned oocyte retrieval seems prudent [92] to reduce risk of anesthesia complications associated with delayed gastric emptying [93, 94].

## Conclusion

Obesity is a major public health challenge with adverse metabolic and reproductive phenotypes. Affected patients face the dual stigmas of obesity and reproductive inadequacy, where fertility loss often induces shame, distress, and low self-esteem. Those seeking infertility treatment with overweight or obesity are at heightened risk of cardiometabolic consequences as well as negatively impacting reproductive outcome with IVF. Weight loss, physical activity, and dietary modification are thus laudable pre-conception interventions for overweight/obese women. Since SG is a potent modifier of insulin status, it sets the metabolic climate for ovarian function and determines reproductive capacity. As a new addition to the therapeutic repertoire in weight management, SG when properly applied, can improve treatment responses for an important subgroup of infertility patients. Yet, its numerous signaling targets involving the human ovary will need to inform future study, and off-label use of SG should be discouraged in the meantime.

## Data Availability

No datasets were generated or analysed during the current study.

## References

[1] Hales CM, Carroll MD, Fryar CD, Ogden CL. Prevalence of obesity and severe obesity among adults: United States, 2017–2018. NCHS Data Brief #360. Hyattsville, MD: National Center for Health Statistics 2020. doi: https://www.cdc.gov/nchs/products/databriefs/db360.htm 32487284

[2] García-Ferreyra J, Carpio J, Zambrano M, Valdivieso-Mejía P, Valdivieso-Rivera P. Overweight and obesity significantly reduce pregnancy, implantation, and live birth rates in women undergoing *in vitro* fertilization procedures. JBRA Assist Reprod. 2021;25(3):394–402. 10.5935/1518-0557.20200105. 33710838 10.5935/1518-0557.20200105 PMC8312282

[3] George JS, Srouji SS, Little SE, Ginsburg ES, Lanes A. The impact of increasing body mass index on in vitro fertilization treatment, obstetrical, and neonatal outcomes. Am J Obstet Gynecol. 2024;230(2):239. 10.1016/j.ajog.2023.10.018. 10.1016/j.ajog.2023.10.018 37852521

[4] Aghaei B, Moradi F, Soleimani D, Moradinazar M, Khosravy T, Samadi M. Glycemic index, glycemic load, dietary inflammatory index, and risk of infertility in women. Food Sci Nutr. 2023;11(10):6413–24. 10.1002/fsn3.3584. 37823091 10.1002/fsn3.3584 PMC10563745

[5] Liu D, Li L, Sun N, Zhang X, Yin P, Zhang W, et al. Effects of body mass index on IVF outcomes in different age groups. BMC Womens Health. 2023;23(1):416. 10.1186/s12905-023-02540-8. 37553621 10.1186/s12905-023-02540-8 PMC10410781

[6] Aydogan Mathyk B, Quaas AM, Obesity. Weighing in on the evidence. J Assist Reprod Genet. 2021;38(2):343–5. 10.1007/s10815-021-02068-6. 33447951 10.1007/s10815-021-02068-6 PMC7884558

[7] Yoo S. Dynamic energy balance and obesity prevention. J Obes Metab Syndr. 2018;27(4):203–12. 10.7570/jomes.2018.27.4.203. 31089565 10.7570/jomes.2018.27.4.203 PMC6513301

[8] Moyad MA. Embracing the pros and cons of the new weight loss medications (Semaglutide, Tirzepatide, Etc.). Curr Urol Rep. 2023: *in press.*10.1007/s11934-023-01180-7 10.1007/s11934-023-01180-7 37659049

[9] Patel PN, Fox CK, Bensignor MO, Bomberg EM. Weight loss from combination anti-obesity medication regimens can approach that achieved from bariatric surgery. JCEM Case Rep. 2023;1(1):luac038. 10.1210/jcemcr/luac038. 37908264 10.1210/jcemcr/luac038 PMC10578411

[10] Nuako A, Tu L, Reyes KJC, Chhabria SM, Stanford FC. Pharmacologic treatment of obesity in reproductive aged women. Curr Obstet Gynecol Rep. 2023;12(2):138–46. 10.1007/s13669-023-00350-1. 37427372 10.1007/s13669-023-00350-1 PMC10328448

[11] Sills ES, Wood SH, Walsh APH. Intraovarian condensed platelet cytokines for infertility and menopause—Mirage or miracle? Biochimie. 2023;204:41–7. 10.1016/j.biochi.2022.08.020. 36075561 10.1016/j.biochi.2022.08.020

[12] Frasca D, Bharath LP, Nikolajczyk B. Editorial: obesity, metabolism and the immune system. Front Aging. 2022;3:1016274. 10.3389/fragi.2022.1016274. 36160605 10.3389/fragi.2022.1016274 PMC9498576

[13] Poljsak B, Kovač V, Milisav I. Healthy lifestyle recommendations: do beneficial effects originate from NAD + amount at the cellular level? Oxid Med Cell Longev. 2020;2020:8819627. 10.1155/2020/8819627. 33414897 10.1155/2020/8819627 PMC7752291

[14] Przygrodzka E, Hou X, Zhang P, Plewes MR, Franco R, Davis JS. PKA and AMPK signaling pathways differentially regulate luteal steroidogenesis. Endocrinology. 2021;162(4):bqab015. 10.1210/endocr/bqab015. 33502468 10.1210/endocr/bqab015 PMC7899060

[15] Froment P, Plotton I, Giulivi C, Fabre S, Khoueiry R, Mourad NI, et al. At the crossroads of fertility and metabolism: the importance of AMPK-dependent signaling in female infertility associated with hyperandrogenism. Hum Reprod. 2022;37(6):1207–28. 10.1093/humrep/deac067. 35459945 10.1093/humrep/deac067

[16] Liu L, Hao M, Zhang J, Chen Z, Zhou J, Wang C, et al. FSHR-mTOR-HIF1 signaling alleviates mouse follicles from AMPK-induced Atresia. Cell Rep. 2023;42(10):113158. 10.1016/j.celrep.2023.113158. 37733588 10.1016/j.celrep.2023.113158

[17] Sills ES, Harrity C, Wood SH, Tan SL. mTOR Inhibition via low-dose, pulsed Rapamycin with intraovarian condensed platelet cytokines: an individualized protocol to recover diminished reserve? J Pers Med. 2023;13(7):1147. 10.3390/jpm13071147. 37511761 10.3390/jpm13071147 PMC10381109

[18] Jurca CM, Kozma K, Petchesi CD, Zaha DC, Magyar I, Munteanu M, et al. Tuberous sclerosis, type II diabetes mellitus and the PI3K/AKT/mTOR signaling pathways: case report and literature review. Genes (Basel). 2023;14(2):433. 10.3390/genes14020433. 36833359 10.3390/genes14020433 PMC9957184

[19] Caron E, Ghosh S, Matsuoka Y, Ashton-Beaucage D, Therrien M, Lemieux S, et al. A comprehensive map of the mTOR signaling network. Mol Syst Biol. 2010;6:453. 10.1038/msb.2010.108. 21179025 10.1038/msb.2010.108 PMC3018167

[20] Guo Z, Yu Q. Role of mTOR signaling in female reproduction. Front Endocrinol (Lausanne). 2019;10:692. 10.3389/fendo.2019.00692. 31649622 10.3389/fendo.2019.00692 PMC6794368

[21] Joo YE, Jeong PS, Lee S, Jeon SB, Gwon MA, Kim MJ, et al. Anethole improves the developmental competence of Porcine embryos by reducing oxidative stress via the Sonic Hedgehog signaling pathway. J Anim Sci Biotechnol. 2023;14(1):32. 10.1186/s40104-022-00824-x. 36814325 10.1186/s40104-022-00824-x PMC9945695

[22] Griffiths HBS, Williams C, King SJ, Allison SJ. Nicotinamide adenine dinucleotide (NAD+): essential redox metabolite, co-substrate and an anti-cancer and anti-ageing therapeutic target. Biochem Soc Trans. 2020;48(3):733–44. 10.1042/BST20190033. 32573651 10.1042/BST20190033

[23] Yang Q, Cong L, Wang Y, Luo X, Li H, Wang H, et al. Increasing ovarian NAD + levels improve mitochondrial functions and reverse ovarian aging. Free Radic Biol Med. 2020;156:1–10. 10.1016/j.freeradbiomed.2020.05.003. 32492457 10.1016/j.freeradbiomed.2020.05.003

[24] Miao Y, Cui Z, Gao Q, Rui R, Xiong B. Nicotinamide mononucleotide supplementation reverses the declining quality of maternally aged oocytes. Cell Rep. 2020;32(5):107987. 10.1016/j.celrep.2020.107987. 32755581 10.1016/j.celrep.2020.107987

[25] Mathew H, Castracane VD, Mantzoros C. Adipose tissue and reproductive health. Metabolism. 2018;86:18–32. 10.1016/j.metabol.2017.11.006. 29155136 10.1016/j.metabol.2017.11.006

[26] Zhang X, Ha S, Lau HC, Yu J. Excess body weight: novel insights into its roles in obesity comorbidities. Semin Cancer Biol. 2023;92:16–27. 10.1016/j.semcancer.2023.03.008. 36965839 10.1016/j.semcancer.2023.03.008

[27] Peluso JJ, Gordon LR. Nonneoplastic and neoplastic changes in the ovary. *In*: Mohr U, Dungworth DL, Capen CC, editors Pathobiology of the aging rat. ILSI, Washington DC 1992:351– 64. doi: https://ntp.niehs.nih.gov/sites/default/files/nnl/female_reproductive/ovary/fatchg/ovary-fatty-change-pdf-508.pdf

[28] Malekpour P, Hasanzadeh R, Javedani Masroor M, Chaman R, Motaghi Z. Effectiveness of a mixed lifestyle program in couples undergoing assisted reproductive technology: A study protocol. Reprod Health. 2023;20(1):112. 10.1186/s12978-023-01652-6. 37528465 10.1186/s12978-023-01652-6 PMC10394976

[29] Yang C, Yang S, Zheng W, Zu R, Ran S, Wu H, et al. Effect of a 60-day weight reduction intervention prior to IVF/ICSI on perinatal outcomes in overweight or obese infertile women. Front Endocrinol (Lausanne). 2022;13:1062790. 10.3389/fendo.2022.1062790. 36531452 10.3389/fendo.2022.1062790 PMC9755661

[30] Mio K, Togo-Ohno M, Tadenuma N, Ogawa R, Yamanaka C, Aoe S. A single administration of barley β-glucan and arabinoxylan extracts reduces blood glucose levels at the second meal via intestinal fermentation. Biosci Biotechnol Biochem. 2022;87(1):99–107. 10.1093/bbb/zbac171. 36307381 10.1093/bbb/zbac171

[31] Suzuki S, Aoe S. High β-glucan barley supplementation improves glucose tolerance by increasing GLP-1 secretion in diet-induced obesity mice. Nutrients. 2021;13(2):527. 10.3390/nu13020527. 33561965 10.3390/nu13020527 PMC7915888

[32] Johannsen DL, Ravussin E. The role of mitochondria in health and disease. Curr Opin Pharmacol. 2009;9(6):780–6. 10.1016/j.coph.2009.09.002. 19796990 10.1016/j.coph.2009.09.002 PMC3951182

[33] John NA, O’Brien LT. Insight into type 2 diabetes impaired exercising mitochondrial oxidative flux: is it blood flow, mitochondria, or neither? J Physiol. 2022;600(4):707–8. 10.1113/JP281551. 33783836 10.1113/JP281551

[34] Gerhart-Hines Z, Rodgers JT, Bare O, Lerin C, Kim SH, Mostoslavsky R, et al. Metabolic control of muscle mitochondrial function and fatty acid oxidation through SIRT1/PGC-1alpha. EMBO J. 2007;26(7):1913–23. 10.1038/sj.emboj.7601633. 17347648 10.1038/sj.emboj.7601633 PMC1847661

[35] Wu D, Yang Y, Hou Y, Zhao Z, Liang N, Yuan P, et al. Increased mitochondrial fission drives the reprogramming of fatty acid metabolism in hepatocellular carcinoma cells through suppression of Sirtuin 1. Cancer Commun (Lond). 2022;42(1):37–55. 10.1002/cac2.12247. 34981667 10.1002/cac2.12247 PMC8753313

[36] Lagouge M, Argmann C, Gerhart-Hines Z, Meziane H, Lerin C, Daussin F, et al. Resveratrol improves mitochondrial function and protects against metabolic disease by activating SIRT1 and PGC-1alpha. Cell. 2006;127(6):1109–22. 10.1016/j.cell.2006.11.013. 17112576 10.1016/j.cell.2006.11.013

[37] Lei MY, Cong L, Liu ZQ, Liu ZF, Ma Z, Liu K, et al. Resveratrol reduces DRP1-mediated mitochondrial dysfunction via the SIRT1-PGC1α signaling pathway in manganese-induced nerve damage in mice. Environ Toxicol. 2022;37(2):282–98. 10.1002/tox.23397. 34738708 10.1002/tox.23397

[38] Li Y, Liu X, Wan L, Han B, Ma S, Pan H, et al. Metformin suppresses cardiac fibroblast proliferation under high-glucose conditions via regulating the mitochondrial complex I protein Grim-19 involved in the Sirt1/Stat3 signaling pathway. Free Radic Biol Med. 2023;206:1–12. 10.1016/j.freeradbiomed.2023.06.013. 37353174 10.1016/j.freeradbiomed.2023.06.013

[39] Shi X, Li L, Liu Z, Wang F, Huang H. Exploring the mechanism of Metformin action in alzheimer’s disease and type 2 diabetes based on network pharmacology, molecular docking, and molecular dynamic simulation. Ther Adv Endocrinol Metab. 2023;14:20420188231187493. 10.1177/20420188231187493. 37780174 10.1177/20420188231187493 PMC10540612

[40] Bahadorani S, Cho J, Lo T, Contreras H, Lawal HO, Krantz DE, et al. Neuronal expression of a single-subunit yeast NADH-ubiquinone oxidoreductase (Ndi1) extends *Drosophila* lifespan. Aging Cell. 2010;9(2):191–202. 10.1111/j.1474-9726.2010.00546.x. 20089120 10.1111/j.1474-9726.2010.00546.x PMC2860002

[41] Oz N, Vayndorf EM, Tsuchiya M, McLean S, Turcios-Hernandez L, Pitt JN, et al. Evidence that conserved essential genes are enriched for pro-longevity factors. Geroscience. 2022;44(4):1995–2006. 10.1007/s11357-022-00604-5. 35695982 10.1007/s11357-022-00604-5 PMC9616985

[42] Amorim JA, Coppotelli G, Rolo AP, Palmeira CM, Ross JM, Sinclair DA. Mitochondrial and metabolic dysfunction in ageing and age-related diseases. Nat Rev Endocrinol. 2022;18(4):243–58. 10.1038/s41574-021-00626-7. 35145250 10.1038/s41574-021-00626-7 PMC9059418

[43] Zhang L, Wu J, Zhu Z, He Y, Fang R, Mitochondrion. A Bridge linking aging and degenerative diseases. Life Sci. 2023;322:121666. 10.1016/j.lfs.2023.121666. 37030614 10.1016/j.lfs.2023.121666

[44] Isola JVV, Zanini BM, Hense JD, Alvarado-Rincón JA, Garcia DN, Pereira GC, et al. Mild calorie restriction, but not 17α-estradiol, extends ovarian reserve and fertility in female mice. Exp Gerontol. 2022;159:111669. 10.1016/j.exger.2021.111669. 35032571 10.1016/j.exger.2021.111669 PMC8920046

[45] Jesús PL, Arenas-Ríos E, Ruíz-Ramos M, Flores-Alonso JC, Mendoza-Núñez VM, Arrieta-Cruz I, et al. Effect of chronic moderate caloric restriction on the reproductive function in aged male Wistar rats. Nutrients. 2022;14(6):1256. 10.3390/nu14061256. 35334913 10.3390/nu14061256 PMC8952234

[46] Haines MS. Endocrine complications of anorexia nervosa. J Eat Disord. 2023;11(1):24. 10.1186/s40337-023-00744-9. 36793059 10.1186/s40337-023-00744-9 PMC9933399

[47] Rashid S, Wong C, Roy R. Developmental plasticity and the response to nutrient stress in *Caenorhabditis elegans*. Dev Biol. 2021;475:265–76. 10.1016/j.ydbio.2021.01.015. 33549550 10.1016/j.ydbio.2021.01.015

[48] Webster AK, Chitrakar R, Taylor SM, Baugh LR. Alternative somatic and germline gene-regulatory strategies during starvation-induced developmental arrest. Cell Rep. 2022;41(2):111473. 10.1016/j.celrep.2022.111473. 36223742 10.1016/j.celrep.2022.111473 PMC9608353

[49] McAuley MT. Dietary restriction and ageing: recent evolutionary perspectives. Mech Ageing Dev. 2022;208:111741. 10.1016/j.mad.2022.111741. 36167215 10.1016/j.mad.2022.111741

[50] Rau V, Flatt T, Korb J. The remoulding of dietary effects on the fecundity / longevity trade-off in a social insect. BMC Genomics. 2023;24(1):244. 10.1186/s12864-023-09335-z. 37147612 10.1186/s12864-023-09335-z PMC10163710

[51] Underwood WJ, McGlone JJ, Swanson J, Anderson KA, Anthony R. Agricultural animal welfare (Chap. 15) *In*: Laboratory Animal Welfare, American College of Laboratory Animal Medicine. Academic Press 2014:233– 78. 10.1016/B978-0-12-385103-1.00015-4

[52] Li C, Zhang H, Wu H, Li R, Wen D, Tang Y, et al. Intermittent fasting reverses the declining quality of aged oocytes. Free Radic Biol Med. 2023;195:74–88. 10.1016/j.freeradbiomed.2022.12.084. 36581058 10.1016/j.freeradbiomed.2022.12.084

[53] Hayashida S, Arimoto A, Kuramoto Y, Kozako T, Honda S, Shimeno H, et al. Fasting promotes the expression of SIRT1, an NAD + dependent protein deacetylase, via activation of PPARalpha in mice. Mol Cell Biochem. 2010;339(1–2):285–92. 10.1007/s11010-010-0391-z. 20148352 10.1007/s11010-010-0391-z

[54] Yang NC, Song TY, Chang YZ, Chen MY, Hu ML. Up-regulation of nicotinamide phosphoribosyltransferase and increase of NAD + levels by glucose restriction extend replicative lifespan of human fibroblast Hs68 cells. Biogerontology. 2015;16(1):31–42. 10.1007/s10522-014-9528-x. 25146190 10.1007/s10522-014-9528-x

[55] Napoleão A, Fernandes L, Miranda C, Marum AP. Effects of calorie restriction on healthspan and insulin resistance: classic calorie restriction diet vs. ketosis-inducing Diet Nutrients. 2021;13(4):1302. 10.3390/nu13041302. 33920973 10.3390/nu13041302 PMC8071299

[56] Aksungar FB, Sarıkaya M, Coskun A, Serteser M, Unsal I. Comparison of intermittent fasting versus caloric restriction in obese subjects: A two year follow-up. J Nutr Health Aging. 2017;21(6):681–5. 10.1007/s12603-016-0786-y.28537332 10.1007/s12603-016-0786-y

[57] Tilly JL, Sinclair DA. Germline energetics, aging, and female infertility. Cell Metab. 2013;17(6):838–50. 10.1016/j.cmet.2013.05.007. 23747243 10.1016/j.cmet.2013.05.007 PMC3756096

[58] Fernandes SA, Demetriades C. The multifaceted role of nutrient sensing and mTORC1 signaling in physiology and aging. Front Aging. 2021;2:707372. 10.3389/fragi.2021.707372. 35822019 10.3389/fragi.2021.707372 PMC9261424

[59] Kim HS, Pickering AM. Protein translation paradox: implications in translational regulation of aging. Front Cell Dev Biol. 2023;11:1129281. 10.3389/fcell.2023.1129281. 36711035 10.3389/fcell.2023.1129281 PMC9880214

[60] Moldakozhayev A, Gladyshev VN. Metabolism, homeostasis, and aging. Trends Endocrinol Metab. 2023;34(3):158–69. 10.1016/j.tem.2023.01.003. 36681595 10.1016/j.tem.2023.01.003 PMC11096277

[61] Orskov C. Glucagon-like peptide-1, a new hormone of the entero-insular axis. Diabetologia. 1992;35(8):701–11. pubmed.ncbi.nlm.nih.gov/1324859/. 1324859

[62] Smith NK, Hackett TA, Galli A, Flynn CR. GLP-1: molecular mechanisms and outcomes of a complex signaling system. Neurochem Int. 2019;128:94–105. 10.1016/j.neuint.2019.04.010. 31002893 10.1016/j.neuint.2019.04.010 PMC7081944

[63] Chaves RN, Duarte AB, Rodrigues GQ, Celestino JJ, Silva GM, Lopes CA, et al. The effects of insulin and follicle-simulating hormone (FSH) during *in vitro* development of ovarian goat preantral follicles and the relative mRNA expression for insulin and FSH receptors and cytochrome P450 aromatase in cultured follicles. Biol Reprod. 2012;87(3):69. 10.1095/biolreprod.112.099010. 22811569 10.1095/biolreprod.112.099010

[64] Kordowitzki P, Krajnik K, Skowronska A, Skowronski MT. Pleiotropic effects of IGF-1 on the oocyte. Cells. 2022;11(10):1610. 10.3390/cells11101610. 35626647 10.3390/cells11101610 PMC9140015

[65] Nagao H, Cai W, Wewer Albrechtsen NJ, Steger M, Batista TM, Pan H, et al. Distinct signaling by insulin and IGF-1 receptors and their extra- and intracellular domains. Proc Natl Acad Sci USA. 2021;118(17):e2019474118. 10.1073/pnas.2019474118. 33879610 10.1073/pnas.2019474118 PMC8092608

[66] Mukherjee D, Majumder S, Moulick SR, Pal P, Mallick B, Chakraborty A, et al. Signaling pathways in insulin- and IGF-I mediated oocyte maturation in lower vertebrates. Indian J Biochem Biophys. 2014;51(6):520–6. https://pubmed.ncbi.nlm.nih.gov/25823225/. 25823225

[67] Kanda R, Hiraike H, Wada-Hiraike O, Ichinose T, Nagasaka K, Sasajima Y, et al. Expression of the glucagon-like peptide-1 receptor and its role in regulating autophagy in endometrial cancer. BMC Cancer. 2018;18(1):657. 10.1186/s12885-018-4570-8. 29907137 10.1186/s12885-018-4570-8 PMC6003019

[68] Violette CJ, Agarwal R, Mandelbaum RS, González JL, Hong KM, Roman LD, et al. The potential role of GLP-1 receptor agonist targeting in fertility-sparing treatment in obese patients with endometrial malignant pathology: A call for research. Expert Rev Anticancer Ther. 2023;23(4):385–95. 10.1080/14737140.2023.2194636. 36944434 10.1080/14737140.2023.2194636

[69] Gomez E, Pritchard C, Herbert TP. cAMP-dependent protein kinase and Ca^2+^ influx through L-type voltage-gated calcium channels mediate Raf-independent activation of extracellular regulated kinase in response to glucagon-like peptide-1 in pancreatic beta-cells. J Biol Chem. 2002;277(50):48146–51. 10.1074/jbc.M209165200. 12364324 10.1074/jbc.M209165200

[70] Cheng CK, Luo JY, Lau CW, Cho WC, Ng CF, Ma RCW, et al. A GLP-1 analog lowers ER stress and enhances protein folding to ameliorate homocysteine-induced endothelial dysfunction. Acta Pharmacol Sin. 2021;42(10):1598–609. 10.1038/s41401-020-00589-x. 33495519 10.1038/s41401-020-00589-x PMC8463564

[71] Sills ES, Genton MG, Perloe M, Schattman GL, Bralley JA, Tucker MJ. Plasma homocysteine, fasting insulin, and androgen patterns among women with polycystic ovaries and infertility. J Obstet Gynaecol Res. 2001;27(3):163–8. 10.1111/j.1447-0756.2001.tb01241.x. 11561833 10.1111/j.1447-0756.2001.tb01241.x

[72] Buteau J, Foisy S, Rhodes CJ, Carpenter L, Biden TJ, Prentki M. Protein kinase Czeta activation mediates glucagon-like peptide-1-induced pancreatic beta-cell proliferation. Diabetes. 2001;50(10):2237–43. 10.2337/diabetes.50.10.2237. 11574404 10.2337/diabetes.50.10.2237

[73] Zhao H, Wang L, Wei R, Xiu D, Tao M, Ke J, et al. Activation of glucagon-like peptide-1 receptor inhibits tumourigenicity and metastasis of human pancreatic cancer cells via PI3K/Akt pathway. Diabetes Obes Metab. 2014;16(9):850–60. 10.1111/dom.12291. 24641303 10.1111/dom.12291

[74] Crittenden SL, Lee C, Mohanty I, Battula S, Knobel K, Kimble J. Sexual dimorphism of niche architecture and regulation of the *Caenorhabditis elegans* germline stem cell pool. Mol Biol Cell. 2019;30(14):1757–69. 10.1091/mbc.E19-03-0164. 31067147 10.1091/mbc.E19-03-0164 PMC6727753

[75] Watanabe JH, Kwon J, Nan B, Reikes A. Trends in glucagon-like peptide 1 receptor agonist use, 2014 to 2022. J Am Pharm Assoc (2003) 2023:S1544-3191(23)00309-6. 10.1016/j.japh.2023.10.002 10.1016/j.japh.2023.10.002 37821008

[76] Chao AM, Tronieri JS, Amaro A, Wadden TA. Semaglutide for the treatment of obesity. Trends Cardiovasc Med. 2023;33(3):159–66. 10.1016/j.tcm.2021.12.008. 34942372 10.1016/j.tcm.2021.12.008 PMC9209591

[77] Dagli N, Kumar S, Ahmad R, Narwaria M, Haque M. An update on semaglutide research: A bibliometric analysis and a literature review. Cureus. 2023;15(10):e46510. 10.7759/cureus.46510. 37808605 10.7759/cureus.46510 PMC10552354

[78] European Medicines Agency. Wegovy (product information) EMA/162766/2023:1–3. doi: https://www.ema.europa.eu/en/documents/overview/wegovy-epar-medicine-overview_en.pdf

[79] Blum D. An extreme risk of taking Ozempic: Malnutrition. The New York Times [*newspaper*] April 21, 2023. doi: nytimes.com/2023/04/21/well/eat/ozempic-side-effects-malnutrition.html

[80] Han SH, Safeek R, Ockerman K, Trieu N, Mars P, Klenke A et al. Public interest in off-label use of glucagon-like peptide 1 agonists (Ozempic) for cosmetic weight loss: A Google trends analysis. Aesthet Surg J 2023:sjad211. 10.1093/asj/sjad211 10.1093/asj/sjad211 37402640

[81] Zhang W, Zhang J. Semaglutide pretreatment induces cardiac autophagy to reduce myocardial injury in septic mice. Discov Med. 2023;35(178):853–60. 10.24976/Discov.Med.202335178.80. 37811623 10.24976/Discov.Med.202335178.80

[82] Iwai S, Kaji K, Nishimura N, Kubo T, Tomooka F, Shibamoto A, et al. Glucagon-like peptide-1 receptor agonist, semaglutide attenuates chronic liver disease-induced skeletal muscle atrophy in diabetic mice. Biochim Biophys Acta Mol Basis Dis. 2023;1869(7):166770. 10.1016/j.bbadis.2023.166770. 37276988 10.1016/j.bbadis.2023.166770

[83] Reis-Barbosa PH, Marcondes-de-Castro IA, Marinho TS, Aguila MB, Mandarim-de-Lacerda CA. The mTORC1/AMPK pathway plays a role in the beneficial effects of semaglutide (GLP-1 receptor agonist) on the liver of obese mice. Clin Res Hepatol Gastroenterol. 2022;46(6):101922. 10.1016/j.clinre.2022.101922. 35427802 10.1016/j.clinre.2022.101922

[84] De Barra C, O’Shea D, Hogan AE. NK cells vs. obesity: A Tale of dysfunction & redemption. Clin Immunol. 2023;255:109744. 10.1016/j.clim.2023.109744. 37604354 10.1016/j.clim.2023.109744

[85] Li Q, Tuo X, Li B, Deng Z, Qiu Y, Xie H. Semaglutide attenuates excessive exercise-induced myocardial injury through inhibiting oxidative stress and inflammation in rats. Life Sci. 2020;250:117531. 10.1016/j.lfs.2020.117531. 32151691 10.1016/j.lfs.2020.117531

[86] Sills ES, Wood SH. Epigenetics, ovarian cell plasticity, and platelet-rich plasma: mechanistic theories. Reprod Fertil. 2022;3(4):C44–51. 10.1530/RAF-22-0078. 36255031 10.1530/RAF-22-0078 PMC9782453

[87] Li Z, Zhang M, Zheng J, Tian Y, Zhang H, Tan Y, et al. Human umbilical cord mesenchymal stem cell-derived exosomes improve ovarian function and proliferation of premature ovarian insufficiency by regulating the Hippo signaling pathway. Front Endocrinol (Lausanne). 2021;12:711902. 10.3389/fendo.2021.711902. 34456868 10.3389/fendo.2021.711902 PMC8397419

[88] Smits MM, Van Raalte DH. Safety of semaglutide. Front Endocrinol (Lausanne). 2021;12:645563. 10.3389/fendo.2021.645563. 34305810 10.3389/fendo.2021.645563 PMC8294388

[89] Novo Nordisk A/S. WEGOVY (semaglutide for subcutaneous injection). Manufacturer’s Package Insert. 2021. https://www.accessdata.fda.gov/drugsatfda_docs/label/2021/215256s000lbl.pdf. doi:.

[90] Anala AD, Saifudeen ISH, Ibrahim M, Nanda M, Naaz N, Atkin SL. The potential utility of Tirzepatide for the management of polycystic ovary syndrome. J Clin Med. 2023;12(14):4575. 10.3390/jcm12144575. 37510690 10.3390/jcm12144575 PMC10380206

[91] Hall S, Isaacs D, Clements JN. Pharmacokinetics and clinical implications of semaglutide: A new glucagon-like peptide (GLP)-1 receptor agonist. Clin Pharmacokinet. 2018;57(12):1529–38. 10.1007/s40262-018-0668-z. 29915923 10.1007/s40262-018-0668-z

[92] Wajtryt O, Dedecjus M. Pregnancy after exposure to GLP-1 receptor agonists: case report and literature review [*abstract*]. 25th European Congress of endocrinology (Istanbul) May 2023. Endocr Abstracts. 2023;90:EP532. 10.1530/endoabs.90.EP532.

[93] Fujino E, Cobb KW, Schoenherr J, Gouker L, Lund E. Anesthesia considerations for a patient on semaglutide and delayed gastric emptying. Cureus. 2023;15(7):e42153. 10.7759/cureus.42153. 37602101 10.7759/cureus.42153 PMC10438952

[94] Sherwin M, Hamburger J, Katz D, DeMaria S Jr. Influence of semaglutide use on the presence of residual gastric solids on gastric ultrasound: A prospective observational study in volunteers without obesity recently started on semaglutide. Can J Anaesth. 2023;70(8):1300–6. 10.1007/s12630-023-02549-5. 37466909 10.1007/s12630-023-02549-5

